# Pharmacogenetic Analysis of the *MIR146A* rs2910164 and *MIR155* rs767649 Polymorphisms and Response to Anti-TNF Treatment in Patients with Crohn’s Disease and Psoriasis

**DOI:** 10.3390/genes14020445

**Published:** 2023-02-09

**Authors:** Paraskevi Nani, Melpomeni Ladopoulou, Evgenia H. Papaioannou, Evangelia D. Papagianni, Charalabos Antonatos, Panagiotis Xiropotamos, Andreas Kapsoritakis, Petros S. Potamianos, Konstantinos Karmiris, Charalambos Tzathas, Aikaterini Patsatsi, Elisavet Lazaridou, Efterpi Zafiriou, Angeliki Roussaki-Schulze, Sophia Georgiou, Katerina Grafanaki, Georgios K. Georgakilas, Yiannis Vasilopoulos

**Affiliations:** 1 Laboratory of Genetics, Section of Genetics, Cell Biology and Development, Department of Biology, University of Patras, 26504 Patras, Greece; 2 Gastroenterology Department, University General Hospital of Larissa, 41110 Larissa, Greece; 3 Gastroenterology Department, “Venizeleio Pananeio” General Hospital of Heraklion, 71409 Heraklion, Greece; 4 Gastroenterology Department, “Tzaneio” General Hospital of Piraeus, 18536 Athens, Greece; 5 2nd Dermatology Department, Medical School, Papageorgiou Hospital, Aristotle University, 56403 Thessaloniki, Greece; 6 BioPsorAD Consortium, 26504 Patras, Greece; 7 Department of Dermatology, University General Hospital Larissa, University of Thessaly, 41334 Larissa, Greece; 8 Dermatology Department, Medical School, University of Patras, 26504 Patras, Greece; 9 Laboratory of Hygiene and Epidemiology, Department of Clinical and Laboratory Research, Faculty of Medicine, University of Thessaly, 38334 Volos, Greece

**Keywords:** psoriasis, Crohn’s disease, pharmacogenetics, anti-TNF, miRNA, response to therapy

## Abstract

The clinical heterogeneity regarding the response profile of the antitumor necrosis factor (anti-TNF) in patients with Crohn’s disease (CD) and psoriasis (PsO) is attributed, amongst others, to genetic factors that influence the regulatory mechanisms which orchestrate the inflammatory response. Here, we investigated the possible associations between the *MIR146A* rs2910164 and *MIR155* rs767649 variants and the response to anti-TNF therapy in a Greek cohort of 103 CD and 100 PsO patients. We genotyped 103 CD patients and 100 PsO patients via the PCR-RFLP method, utilizing the de novo formation of a restriction site for the SacI enzyme considering the *MIR146A* rs2910164, while Tsp45I was employed for the *MIR155* rs767649 variant. Additionally, we investigated the potential functional role of the rs767649 variant, exploring in silico the alteration of transcription factor binding sites (TFBSs) mapped on its genomic location. Our single-SNP analysis displayed a significant association between the rare rs767649 A allele and response to therapy (Bonferroni-corrected *p* value = 0.012) in patients with PsO, a result further enhanced by the alteration in the IRF2 TFBS caused by the above allele. Our results highlight the protective role of the rare rs767649 A allele in the clinical remission of PsO, implying its utilization as a pharmacogenetic biomarker.

## 1. Introduction

Tumor Necrosis Factor α (TNFα), a proinflammatory cytokine regulating the NF-κB pathway activity, has been associated with numerous autoimmune diseases such as Crohn’s disease (CD) and psoriasis (PsO) [1,2,3]. CD is a member of the inflammatory bowel diseases (IBD) along with ulcerative colitis (UC) and is a chronic inflammatory disease of the gastrointestinal tract, displaying a variable epidemiological profile according to the geographical location, affecting approximately 3–20/100,000 individuals annually [4,5]. The cutaneous inflammation present in PsO forms distinct erythematous epidermic plaques and leads to the accumulation of hyperproliferative keratinocytes; the prevalence of PsO ranges between 1.83 and 5.32% of the global population [3]. Given the predominant role of TNFα in the pathogenesis of both diseases, anti-TNF monoclonal agents have been established in the therapeutic armamentarium of such autoimmune disorders [6]. A plethora of anti-TNF pharmaceutical approaches have been already approved for the treatment of PsO and CD and established in the clinical routine, including Adalimumab, Infliximab and Etanercept, whereas Certolizumab Pegol and Golimumab are in phase 3 clinical trials regarding PsO. Adalimumab and Golimumab are fully humanized monoclonal antibodies, in contrast to Infliximab which is a recombinant chimeric (75% human, 25% mouse) immunoglobulin G1 monoclonal antibody. Similarly, Certolizumab pegol is a humanized PEGylated monoclonal antibody that lacks the fragment crystallizable (FC) region, while Etanercept is the only nonmonoclonal antibody, exhibiting its TNF-inhibitory action through a soluble fusion protein that consists of the soluble p75 receptor that blocks TNF signaling. All the above biological therapies usually target both soluble and membrane-bound forms of TNF, inhibiting its function [7,8]. Despite the significant efficacy of anti-TNF agents that target the above cytokine in the remission of CD and PsO patients, a significant number fail to achieve clinical remission, developing adverse drug reactions, a clinical heterogeneity that could be partially explained by the interindividual genetic variability as shown by multiple pharmacogenetic studies [9,10,11].

The multilayered complexity of such traits lies in the interactions between environmental and genetic factors, bridged by the epigenetic and post-transcriptional modifications in the inflammatory state [12,13,14,15]. A major example of the above modifications includes micro-RNAs (miRNAs), which are small single-stranded noncoding RNAs that exert post-transcriptional regulation via RNA-induced silencing complexes (RISCs) and have been associated with the pathogenesis of multiple inflammatory disorders such as rheumatoid arthritis, Sjorgen syndrome, multiple sclerosis, CD and PsO [3,15,16,17,18,19,20]. Extensive research interest in the perturbation of these molecules in complex features has illustrated the involvement of several miRNAs in the dysregulated expression patterns of important disease-associated pathways, including NF-κB. MiR-146a and miR-155 are both considered as central regulators of the inflammatory state due to their pleiotropic targeting mechanisms present in numerous cell types [21,22]. Specifically, miR-146a, located on chromosome 5q33, binds to the 3′-UTR of TNF signal transductor *TRAF6* and interleukin 1 (IL-1) receptor kinase *IRAK1* which participate in the NF-κB pathway, resulting in the suppression of NF-κΒ target gene expression. In particular, the signaling transduction derived from the TNF receptor superfamily and Toll/IL-1 family is facilitated by TRAF6. In addition, this molecule acts in concert with the additional mir-146a target *IRAK1* to activate the IkappaB kinase (IKKβ); IRAK1 further enhances the phosphorylation of the p65 subunit as well as its binding to the NF-κB target genes [21]. Similarly, miR-155, located at the long arm of chromosome 21, inhibits the expression of the suppressor of cytokine signaling *SOCS1*, a negative regulator of the JAK/STAT pathway and an inhibitor of IL-17 [3,23]. Specifically, SOCS1 is a central inhibitor of the TLR signaling pathway, binding to the Mal/TIRAP complex and leading to its degradation as well as to IRAK1, modulating its activity; the downregulation of this inhibitory factor via the indirect upregulation of mir-155 has been associated with multiple autoimmune diseases [22]. Nevertheless, expression changes in both miRNAs due to genetic variants located in enhancer/promoter regions (*MIR155* rs767649 regions), or single-nucleotide polymorphisms (SNPs) affecting their functional role (*MIR146A* rs2910164), could hinder their post-transcriptional action, forming a dysregulated cascade [24,25]. Both variants have been associated with PsO and CD, with the *MIR146A* rs2910164 C allele being associated with its underexpression and the *MIR155* rs767649 A allele displaying a tissue-specific expression pattern; however, their putative participation in the response to therapy has not been systematically explored [26,27]. In this study, we conducted a pharmacogenetic analysis of *MIR146A* rs2910164 and *MIR155* rs767649 in response to anti-TNF treatment in Greek patients with CD and PsO.

## 2. Materials and Methods

### 2.1. Clinical Assessment of the Included Patients

Our multidisease cohort consisted of 103 and 100 Greek patients with CD and PsO naïve to anti-TNF therapy, respectively. The CD cohort utilized in this study has been adequately described elsewhere [28]. Briefly, the infliximab treatment was administered intravenously with a 5 mg/kg dose at weeks 0, 2 and 6 and then 5 mg/kg every 8 weeks (depending on response); 80–160 mg of adalimumab were administered subcutaneously at week 0, 40–80 mg at week 2, and then 40 mg every 2 weeks (depending on response). Overall, 103 patients (49 male, 54 female)—derived from 3 different Greek General Hospitals—with concomitant infectious diseases were excluded from the study. Disease activity as well as response to the 24-month anti-TNF therapy were evaluated using the Crohn’s Disease Activity Index (CDAI). Patients presenting a reduction in the CDAI ≥ 70 points were classified as responders, according to the European Crohn’s and Colitis organization guidelines after anti-TNF therapy (Infliximab or Adalimumab) [28]. Considering the PsO cohort, all patients suffered from PsO vulgaris, and the patients’ samples were collected from the Dermatology Departments of the University General Hospital of Larissa and the General University Hospital of Patras as well as the Second Dermatology Department of the Papageorgiou Hospital, Aristotle University, Greece. PsO patients received anti-TNF therapy, namely, Infliximab (53 patients), Adalimumab (10 patients) or Etanercept (37 patients) for 6 months, with a decrease in the Psoriasis Area Severity Index (PASI) greater than 75% (PASI 75) classifying them as responders to the anti-TNF therapy [10].

Written informed consent for participation in the study was obtained from each respective patient, along with the approval from the local ethical committees.

### 2.2. Genotyping

Genomic DNA (gDNA) was extracted from samples of whole peripheral blood obtained during the clinical routine examination of the patient. The samples were collected in coagulant K3-EDTA tubes and processed as per the conventional phenol/chloroform extraction protocol. Genotyping of *MIR146A* rs2910164 and *MIR155* rs767649 SNPs was performed via the polymerase chain reaction–restriction fragment length polymorphism (PCR-RFLP) analysis.

Specific forward and reverse primers for each SNP are displayed in Table 1 along with their respective melting temperatures (Tm) and the incorporated restriction enzyme. Primers utilized for the rs2910164 genotyping formed a restriction site for the *SacI* enzyme, fostered by the presence of the rs2910164 C allele. For *MIR146A* rs2910164, the typical PCR protocol consisted of 1× Taq polymerase and 1× of the respective buffer with 1.5 mM MgCl_2_, 0.1 mM dNTP mix and 25 μM of each primer along with the template gDNA. The cycling protocol included preincubation at 95 °C for 5 min followed by 35 cycles of denaturation at 94 °C for 30 s, annealing at 65 °C for 30 s, extension at 72 °C for 30 s, and a final extension at 72 °C for 5 min. For *MIR155* rs767649, the PCR protocol consisted of 1× Taq polymerase and 1× of the respective buffer with 1.5 mM MgCl_2_, 0.1 mM dNTP mix and 25 μM of each primer with the template gDNA. Cycling protocol included preincubation at 95 °C for 5 min followed by 35 cycles of denaturation at 94 °C for 30 s, annealing at 59 °C for 30 s, extension at 72 °C for 30 s, and a final extension at 72 °C for 5 min. All PCR reactions were conducted using the C1000 Touch Thermal Cycler (BIO-RAD, Hercules, CA, USA). The digestion reactions were performed using 2 units of *SacI* restriction enzyme for *MIR146A* and 1 unit of *Tsp45I* for *MIR155* for 16 h. For *MIR146A* rs2910164, the expected PCR product was 147 bp whereas the digested fragments of the C allele were 122 bp and 25 bp. For *MIR155* rs767649, the uncut product was 294 bp and the cut products were 252 bp and 42 bp for the A allele and 158 bp, 94 bp and 42 bp for the T allele. The evaluation of the derived fragment lengths was conducted using electrophoresis on 3% agarose gel [29,30].

### 2.3. TFBS Analysis on MIR155 rs767649

A motif occurrence analysis was applied to elucidate the implications of *MIR155* rs767649 on transcription factor binding and consequently on the putative miR-155 expression regulation. Two subsequences of the human reference genome (GRCh38) pertaining to the same area, 5000 nucleotides long, in the presence or absence of the polymorphism, along with experimentally derived transcription factor weight matrices derived from JASPAR, were used as the input for the Fimo algorithm [31,32].

### 2.4. Statistical Analysis

Each genetic variant under study was, at first, evaluated for the Hardy–Weinberg equilibrium via the chi-square test. The association between the response to therapy in both diseases, according to the predefined clinical criteria, was assessed with 2 × 2 contingency tables. We explored the putative association between the allele frequencies of *MIR146A* rs2910164 and *MIR155* rs767649 and the response to drug regimens in both our PsO and CD cohorts, grouping all anti-TNF approaches, as performed in large anti-TNF pharmacogenetic studies, as well as drug subgroup analyses [33,34,35,36]. A Cochran–Armitage trend test, allelic and genotypic tests, and tests of dominant or recessive SNP inheritance were utilized to determine the association of *MIR155* rs767649 and *MIR146A* rs2910164 with therapeutic response. Single SNP-analysis-derived *p* values were corrected with the Bonferroni method. The threshold for all statistical tests was set at *p* < 0.05. All statistical analyses were performed using Stata 13.1 (Stata Corp, College Station, TX, USA).

## 3. Results

### 3.1. Clinical Characteristics of the Included Patients

The clinical characteristics of CD and PsO patients are summarized in Table 2. The mean age in the CD cohort ranged between 27.67 and 58.33 years, similarly to the PsO cohort (45.12 ± 11.23). According to the predefined clinical criteria, 71 (68.9%) of the 103 CD patients were evaluated as responders to therapy with a CDAI score reduction of more than 70 according to the European Crohn’s and Colitis organization guidelines [28], whereas 32 patients (31.1%) were classified as nonresponders. Accordingly, 68 (68%) PsO patients were positive responders to the anti-TNF therapy based on the reduction in the PASI score being greater than 75% [10], with 24 patients receiving Etanercept, 41 receiving Infliximab and 3 receiving Adalimumab treatment. Among the nonresponder patients in the PsO cohort, 13 were treated with Etanercept, 12 with Infliximab and 7 with Adalimumab. Out of 71 responders in the CD cohort, 15 were treated with Adalimumab and 56 with Infliximab. The 32 nonresponders were administered either Adalimumab (4) or Infliximab (28).

### 3.2. Genotyping Results

We examined the association of each variant and response to all anti-TNF agents in each disease cohort as well as for each biological agent individually. In the CD cohort, no statistically significant association was observed with respect to both *MIR146A* rs2910164 (*p* = 0.321, *p* corrected (*P_c_*) > 0.99) and the *MIR155* rs767649 (*p* = 0.501, *P_c_* > 0.99) variants (Table 3). The analyses of treatment subgroups, including Adalimumab (Table S1) and Infliximab (Table S2), were unable to further illustrate any significant association (*P_c_* > 0.99).

In contrast to the lack of associations in the CD cohort, the PsO responders displayed a significantly higher frequency of the *MIR155* rs767649 rare A allele and positive response to therapy in the allele-based model of inheritance (odds ratio (95%CI): 0.264 (0.105–0.662), *P_c_* = 0.012) (Table 4). Additional associations were depicted with the genotypic (*P_c_* = 0.008) and the recessive (*P_c_* = 0.004) models of inheritance, further enhancing the protective role of the rare A allele in therapeutic response. Despite the initial significant association (*p* = 0.035), the common G allele of *MIR146A* rs2910164 failed to maintain its statistical significance after Bonferroni correction (*P_c_* = 0.142). However, similar to the CD cohort, no associations were obtained when each drug was examined separately in the PsO cohort, including Etanercept (Table S3), Infliximab (Table S4) and Adalimumab (Table S5).

### 3.3. Motif Analysis Uncovers the Functional Role of MIR155 rs767649

**Figure 1:**
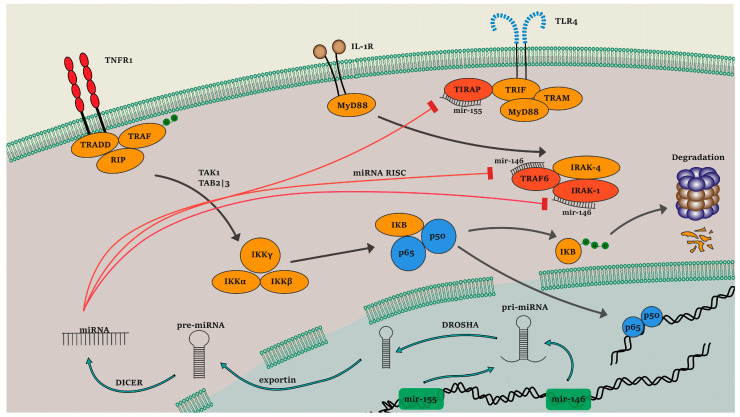
Expression of mir-146a and mir-155 and their implication in the NF-κB pathway. Briefly, the canonical biogenesis pathway of mature miRNAs involves several steps, beginning with their transcriptional regulation, orchestrated via Transcription Factors as is the case with the IRF2 binding to the TFBS harboring the *MIR155* gene. Primary miRNA transcripts (pri-miRNAs) are then processed from the Drosha enzyme into precursor miRNAs (pre-miRNAs) and are exported through Exportin 5 into the cytoplasm. The RNase III endonuclease Dicer cleaves the terminal loop and forms the mature miRNA transcripts [21]. Mir-146a is predicted to bind to the 3′ untranslated region of TNF-receptor associated factor 6 (*TRAF6*) and IL-1 receptor-associated kinase 1 (*IRAK1*) genes, while mir-155 binds to the 3′ untranslated region of the *TIRAP*’s inhibitor, suppressor of cytokine signaling *SOCS1* [21,22].

The transcription factor motif analysis based on JASPAR weight matrices and Fimo highlighted the putatively functional role of *MIR155* rs767649 through in silico analysis [31,32]. Specifically, the rs767649 T to A transversion significantly alters the TFBS of IRF2, with the T allele establishing strong binding (*p* = 2.33 × 10^−5^) in contrast to the A allele that essentially eliminates the probability for IRF2 binding (Figure 1). Our analysis also predicted the binding of SOX21 with both T (*p* = 2.06 × 10^−5^) and A (*p* = 1.59 × 10^−5^) alleles.

## 4. Discussion

Pharmacogenetics, as a field, explores the interindividual variability during the clinically heterogeneous response to therapy. The genetic variation mapped to noncoding regions, including enhancer/promoter regions as well as transcriptional regulatory elements such as mi-RNAs, might be able to additionally explain the vast discrepancies during the response to drug regimens, thus participating in the expanding field of personalized medicine. Here, we investigated the potential pharmacogenetic role of *MIR155* rs767649 and *MIR146A* rs2910164 genetic variants considering the response to anti-TNF therapy in two inflammatory disorders with similar pathogenic mechanisms, PsO and CD. To our knowledge, this is the first pharmacogenetic study that explores putative associations of *MIR155* rs767649 with response to anti-TNF therapy in patients with CD and PsO, while *MIR146A* rs2910164 SNP has been previously studied in only a Greek CD cohort. For this purpose, 103 CD patients and 100 PsO patients of Greek origin were genotyped for both variants using the PCR-RFLP method, creating a de novo restriction site for the *SacI* endonuclease regarding the *MIR146A* rs2910164 SNP genotyping, whilst the *Tsp45I* enzyme was utilized for the *MIR155* rs767649 PCR-RFLP analysis.

The common G allele of the *MIR146A* rs2910164 SNP was associated with a positive response to anti-TNF therapy only in the PsO cohort when grouped together, while the association failed to be maintained after Bonferroni correction (*P_c_* = 0.142) (Table 4). MiR-146a is an important regulator of innate immunity, which has recently been confirmed using knock-out mouse models showing the chronic dysregulation of the NF-κB pathway through the inhibition of *IRAK1* and *TRAF6* mRNAs [3,21]. The rs2910164 variant has depicted its functional role through multiple case–control studies in PsO, as summarized in a recent meta-analysis that uncovered a significant association between the rare CC genotype and lower PsO risk, without, however, additional subgroup analyses according to ethnic background [37]. Regarding CD patients, a similar pharmacogenetic study was conducted by Papaconstantinou et al. in a Greek CD cohort, where no significant associations were found under any model of inheritance [27]. Notably, out of the 100 CD patients included in their study, no CC genotype carriers were found, in accordance with our genotypic results. Specifically, of the 203 Greek patients suffering from PsO and CD in our multidisease cohort, only a single nonresponder to etanercept PsO patient was identified (Supplementary Table S3), further supporting the protective role of the CC genotype in the inflammatory disease onset through altered miR-146a expression levels [37].

Contrary to *MIR146A* rs2910164 where no significant associations were found after correction for multiple comparisons, *MIR155* rs767649 depicted statistically significant associations in the PsO cohort (Table 4), which were further maintained after the applied Bonferroni correction (OR, 95%CI: 0.264 (0.105–0.662); *p* = 0.003, *P_c_* = 0.012) (Table 4) when grouped together. Therefore, the rare A allele of *MIR155* rs767649 SNP displays a protective role in response to anti-TNF therapy (Table 4), driven by alterations in the IRF2 TFBS, as depicted via our bioinformatic analyses (Figure 1). Notably, IRF2 belongs to the PSORS3 Psoriasis Susceptibility locus on the 4q25 chromosome as identified through genetic linkage studies [38]. Contrary to the inheritable risk IRF2 variations pose to PsO susceptibility, its deregulation pattern has been confirmed in the peripheral blood of CD patients, which could be potentially induced by the inflammatory phenotype and not establish a causal association between *IRF2*’s expression pattern and disease onset [39]. Hence, our results could indirectly enhance the genetic role of the regulatory axis *IRF2*-*MIR155* variation in PsO patients regarding the genetic predisposition in both the disease (as identified through previous linkage studies) as well as response to therapy [38]. However, the precise transcriptional activity of the rs767649 SNP in the *MIR155* expression has not yet been elucidated, displaying a cell-specific expression pattern [40,41,42,43]. Despite the molecular heterogeneity, our results highlight the role of the rs767649 polymorphism in the response to anti-TNF therapy, implying an underexpression of miR-155 and, thus, the significant overexpression of the cytokine suppressor gene *SOCS1* (Figure 1).

Nevertheless, several limiting factors are present in our study. Despite the representative PsO and CD patients receiving anti-TNF therapy in the general Greek population, our results need further validation in larger cohorts. In addition, our relatively small sample size hindered the statistical power of our subgroup analyses, failing to show any significant association in contrast to our main results (Table 4; Tables S1–S5). Furthermore, the relatively low minor allele frequency (MAF) of *MIR146A* rs2910164 in our multidisease cohort (MAF = 0.036), in contrast to the European population estimated at around 0.25, hindered the uncovering of its putative role as a pharmacogenetic biomarker. However, this result is in agreement with the MAF reported by Papaconstantinou et al. (MAF = 0.015), in contrast to case–control studies conducted in the Greek population, implying the protective role of the rare C allele in the onset and progression of autoimmune inflammatory diseases such as CD and PsO [44,45]. Furthermore, the nonsignificant associations obtained from our CD cohort analysis could be correlated with NF-κB activity during CD and response to therapy; Han et al. evaluated NF-κB expression through immunohistochemistry and identified clinical subgroups according to the transcription factor’s activity [46]. Therefore, the stratification of CD patients according to the miR-155-associated NF-κB expression could unveil putative pharmacogenetic associations that explain the interindividual variability in clinical remission.

## 5. Conclusions

To the best of our knowledge, we conducted the first pharmacogenetic study on the potential role of *MIR155* rs767649 in response to anti-TNF therapy in patients with PsO, further confirming the lack of association between *MIR146A* rs2910164 and the response to therapy in patients with CD. We highlighted the protective role of the rs767649 rare A allele in the clinical remission of PsO after correction for multiple comparisons (Table 4), a result explained by its functional role as revealed through our bioinformatic analyses. Future studies should not only focus on validating the above results and classifying CD patients in terms of NF-κB activity, but also on depicting the potential pharmacogenetic role of other disease-related variants implicated in regulatory elements that orchestrate the perturbed pathogenic expression.

## Figures and Tables

**Table 1:** Forward and reverse primer sequences and restriction enzymes for the *MIR155* rs767649 and *MIR146A* rs2910164 SNPs PCR-RFLP genotyping.

	Primer Sequence	Tm	Restriction Enzyme
***MIR155* rs767649**			
**Forward primer**	5′-CCTGTATGACAAGGTTGTGTTTG-3′	58.9 °C	*Tsp45I*
**Reverse primer**	5′-GCTGGCATACTATTCTACCCATAA-3′	59.3 °C	
***MIR146A* rs2910164**			
**Forward primer**	5′-CATGGGTTGTGTCAGTGTCAGAGCT-3′	64.6 °C	*SacI*
**Reverse primer**	5′-TGCCTTCTGTCTCCAGTCTTCCAA-3′	62.7 °C	

**Table 2:** Clinical activity index at baseline and clinical activity index after 6 months of treatment for psoriasis and 24 months for Crohn’s disease patients.

Variable	Psoriasis (n = 100)	Crohn’s Disease (n = 103)
Age in years, mean ± S.D.	45.12 ± 11.23	43 ± 15.33
Disease duration in years, mean ± S.D.	15.11 ± 10.35	6.54 ± 3.1
Age of onset in years, mean ± S.D.	38.25 ± 10.75	39 ± 11.25
Clinical index at baseline, mean ± S.D.	28.85 ± 9.65	171.14 ± 114.17
Clinical index after the duration of the therapy, mean ± S.D.	4.27 ± 4.52	48.57 ± 47.82
Responders, percentage	68 (68%)	71 (68.9%)
Nonresponders, percentage	32 (32%)	32 (31.1%)
Anti-TNF therapy (R/NR)		
Etanercept	37 (24/13)	-
Infliximab	53 (41/12)	84 (56/28)
Adalimumab	10 (3/7)	19 (15/4)

Abbreviations: S.D., standard deviation; R, responders; NR, nonresponders.

**Table 3:** Association between the studied genetic variants and response to anti-TNF therapy in the CD cohort.

Gene, Variant	Statistical Test	R	NR	OR	95% CI	P	P_C_
*MIR146A* rs2910164	Genotypic [GG/GC/CC]	68/3/0	29/3/0	-	-	0.507	>0.99
	Cochran–Armitage [G/C]	139/3	61/3	2.278	0.447–11.611	0.321	>0.99
	Dominant [(GG + GC)/CC]	71/0	32/0	2.2	0.042–113.332	0.695	>0.99
	Recessive [GG/(GC + CC)]	68/3	29/3	2.344	0.446–12.310	0.313	>0.99
*MIR155* rs767649	Genotypic [TT/TA/AA]	29/32/10	13/18/1	-	-	0.217	0.870
	Cochran–Armitage [T/A]	90/52	44/20	0.786	0.419–1.476	0.454	>0.99
	Dominant [(TT + TA)/AA]	61/10	31/1	0.196	0.024–1.607	0.129	0.517
	Recessive [TT/(TA + AA)]	29/42	13/19	1.009	0.431–2.359	0.983	>0.99

Abbreviations: R, responders; NR, nonresponders; OR, odds ratio; CI, confidence intervals. Statistically significant *p* values are denoted in bold.

**Table 4:** Association between the studied genetic variants and response to anti-TNF therapy in the PsO cohort.

Gene, Variant	Statistical Test	R	NR	OR	95% CI	P	P_C_
*MIR146A* rs2910164	Genotypic [GG/GC/CC]	65/3/0	27/4/1	-	-	0.107	0.430
	Cochran–Armitage [G/C]	133/3	58/6	4.586	1.108–18.970	**0.035**	0.142
	Dominant [(GG + GC)/CC]	68/0	31/1	6.523	0.258–164.649	0.254	>0.99
	Recessive [GG/(GC + CC)]	65/3	27/5	4.012	0.895–17.983	0.069	0.278
*MIR155* rs767649	Genotypic [TT/TA/AA]	30/37/1	26/6/0	-	-	**0.002**	**0.008**
	Cochran–Armitage [T/A]	97/39	58/6	0.257	0.102–0.644	**0.003**	**0.012**
	Dominant [(TT + TA)/AA]	67/1	32/0	0.692	0.027–17.464	0.823	>0.99
	Recessive [TT/(TA + AA)]	30/38	26/6	0.182	0.066–0.499	**0.001**	**0.004**

Abbreviations: R, responders; NR, nonresponders; OR, odds ratio; CI, confidence intervals. Statistically significant *p* values are denoted in bold.

## Data Availability

Data are available upon request from the corresponding author.

## Supplementary Materials

The following supporting information can be downloaded at: https://www.mdpi.com/article/10.3390/genes14020445/s1, Table S1: Association between the under-study genetic variants and response to Adalimumab in the CD cohort.; Table S2: Association between the under-study genetic variants and response to Infliximab in the CD cohort.; Table S3: Association between the under-study genetic variants and response to Etanercept in the PsO cohort.; Table S4: Association between the under-study genetic variants and response to Infliximab in the PsO cohort.; Table S5: Association between the under-study genetic variants and response to Adalimumab in the PsO cohort.
