# Supplementary material for: Pharmacogenetic Analysis of the MIR146A rs2910164 and MIR155 rs767649 Polymorphisms and Response to Anti-TNF Treatment in Patients with Crohn’s Disease and Psoriasis
Source: Genes (Basel). 2023 Feb 9;14(2):445. doi: 10.3390/genes14020445 (PMC9957441; doi:10.3390/genes14020445)
Supplement: Supplementary file 1 [file genes-14-00445-s001.zip › genes-2108310-supplementary.pdf]

Supplementary Table 1. Association between the under-study genetic variants and response to Adalimumab in the CD cohort.

Gene, Variant	Statistical Test	Responders	Non-Responders	OR	95% CI	P	P_C
MIR146A rs2910164	Genotypic [GG/GC/CC]	14/1/0	4/0/0	-	-	0.840	>0.99
	Cochran–Armitage [G/C]	29/1	8/0	1.156	0.043-31.064	0.930	>0.99
	Dominant [(GG + GC)/CC]	15/0	4/0	3.444	0.059-199.304	0.550	>0.99
	Recessive [GG/(GC + CC)]	14/1	4/0	1.074	0.036-31.260	0.966	>0.99
MIR155 rs767649	Genotypic [TT/TA/AA]	4/8/3	2/2/0	-	-	0.508	>0.99
	Cochran–Armitage [T/A]	16/14	6/2	0.381	0.066-2.200	0.280	>0.99
	Dominant [(TT + TA)/AA]	12/3	4/0	0.396	0.017-9.288	0.565	>0.99
	Recessive [TT/(TA + AA)]	4/11	2/2	0.363	0.037-3.518	0.382	>0.99

Supplementary Table 2. Association between the under-study genetic variants and response to Infliximab in the CD cohort.

Gene, Variant	Statistical Test	Responders	Non-Responders	OR	95% CI	P	P_C
MIR146A rs2910164	Genotypic [GG/GC/CC]	54/2/0	25/3/0	-	-	0.394	>0.99
	Cochran–Armitage [G/C]	110/2	53/3	3.113	0.504-19.195	0.221	0.884
	Dominant [(GG + GC)/CC]	56/0	28/0	1.982	0.038-102.526	0.733	>0.99
	Recessive [GG/(GC + CC)]	54/2	25/3	3.240	0.508-20.626	0.213	0.852
MIR155 rs767649	Genotypic [TT/TA/AA]	25/24/7	12/15/1	-	-	0.361	>0.99
	Cochran–Armitage [T/A]	74/38	39/17	0.848	0.425-1.694	0.642	>0.99
	Dominant [(TT + TA)/AA]	49/7	27/1	0.259	0.030-2.219	0.217	0.868
	Recessive [TT/(TA + AA)]	25/31	12/16	1.075	0.430-2.685	0.876	>0.99

Supplementary Table 3. Association between the under-study genetic variants and response to Etanercept in the PsO cohort.

Gene, Variant	Statistical Test	Responders	Non-Responders	OR	95% CI	P	P_C
MIR146A rs2910164	Genotypic [GG/GC/CC]	23/1/0	11/1/1	-	-	0.340	>0.99
	Cochran–Armitage [G/C]	47/1	23/3	6.130	0.603-62.226	0.125	0.500
	Dominant [(GG + GC)/CC]	24/0	12/1	4.083	0.127-130.485	0.426	>0.99
	Recessive [GG/(GC + CC)]	23/1	11/2	4.181	0.341-51.237	0.263	>0.99
MIR155 rs767649	Genotypic [TT/TA/AA]	13/11/0	11/2/0	-	-	0.206	0.824

	Cochran–Armitage [T/A]	37/11	24/2	0.280	0.057-1.377	0.117	>0.99
	Dominant [(TT + TA)/AA]	24/0	13/0	1.814	0.034-96.725	0.768	>0.99
	Recessive [TT/(TA + AA)]	13/11	11/2	0.214	0.0388-1.184	0.077	>0.99

Supplementary Table 4. Association between the under-study genetic variants and response to Infliximab in the PsO cohort.

Gene, Variant	Statistical Test	Responders	Non-Responders	OR	95% CI	P	P_C
MIR146A rs2910164	Genotypic [GG/GC/CC]	39/2/0	9/3/0	-	-	0.085	0.340
	Cochran–Armitage [G/C]	80/2	21/3	5.714	0.896-36.438	0.065	0.260
	Dominant [(GG + GC)/CC]	41/0	12/0	3.320	0.062-176.028	0.553	>0.99
	Recessive [GG/(GC + CC)]	39/2	9/3	6.50	0.943-44.802	0.057	0.228
MIR155 rs767649	Genotypic [TT/TA/AA]	14/26/1	8/4/0	-	-	0.126	0.504
	Cochran–Armitage [T/A]	54/28	20/4	0.385	0.120-1.238	0.109	0.438
	Dominant [(TT + TA)/AA]	40/1	12/0	1.080	0.041-28.214	0.963	>0.99
	Recessive [TT/(TA + AA)]	14/27	8/4	0.259	0.066-1.013	0.052	0.208

Supplementary Table 5. Association between the under-study genetic variants and response to Adalimumab in the PsO cohort.

Gene, Variant	Statistical Test	Responders	Non-Responders	OR	95% CI	P	P_C
MIR146A rs2910164	Genotypic [GG/GC/CC]	3/0/0	7/0/0	-	-	0.085	0.340
	Cochran–Armitage [G/C]	6/0	14/0	0.448	0.008-25.162	0.065	0.260
	Dominant [(GG + GC)/CC]	3/0	7/0	0.466	0.007-28.734	0.553	>0.99
	Recessive [GG/(GC + CC)]	3/0	7/0	0.466	0.007-28.734	0.057	0.228
MIR155 rs767649	Genotypic [TT/TA/AA]	3/0/0	7/0/0	-	-	0.880	>0.99
	Cochran–Armitage [T/A]	6/0	14/0	0.448	0.008-25.162	0.696	>0.99
	Dominant [(TT + TA)/AA]	3/0	7/0	0.466	0.007-28.734	0.716	>0.99
	Recessive [TT/(TA + AA)]	3/0	7/0	0.466	0.007-28.734	0.716	>0.99
